# Supplementary material for: Single cell transcriptomic analysis of the immune cell compartment in the human small intestine and in Celiac disease
Source: Nat Commun. 2022 Aug 22;13:4920. doi: 10.1038/s41467-022-32691-5 (PMC9395525; doi:10.1038/s41467-022-32691-5)
Supplement: Supplementary file 1 — Supplementary Information [file 41467_2022_32691_MOESM1_ESM.pdf]

Title: Single cell transcriptomic analysis of the immune cell compartment in the human small intestine and in Celiac disease

Supplementary Fig. 1

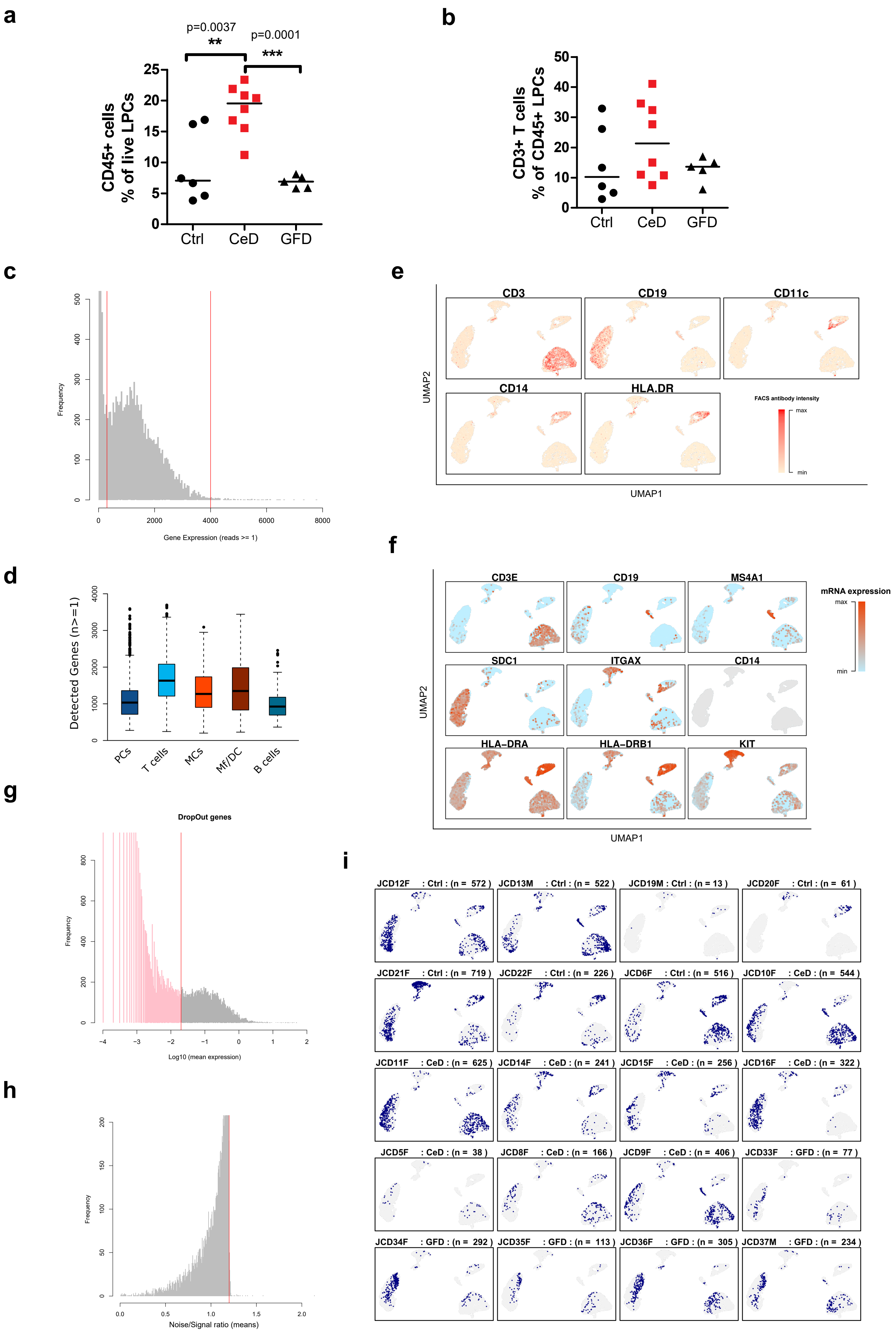

Supplementary Fig. 1 | Single-cell CD45$^+$ immune cells in human small intestine in Ctrl, CeD and GFD samples.

a Scatter plots of the proportion of the CD45$^+$ cells of live Lamina Propria (LP) derived cells (n = 6 (Ctrl), 8 (CeD), 5 (GFD) donors) (Ctrl = control subjects, CeD = Celiac disease patients and GFD = Celiac patients on gluten-free diet) (two-tailed Student's t-test p values, * < 0.05, ** < 0.01, *** < 0.001). **b** Scatter plots of the percentages of the CD3$^+$ cells in LP among the CD45$^+$ cells between the conditions (n = 6 (Ctrl), 8 (CeD), 5 (GFD) donors). **c** Histogram of the total number of the expressed genes (count reads >= 1) across single cells (red lines are the applied thresholds in including cells (300-4000 genes). **d** Boxplots of the total number of detected genes per UMAP cluster (cell type) (center line, median; box limits, upper and lower quartiles; whiskers, minimum and maximum values; points, outliers, 1.5x interquartile range; n = 2655 (PC), 2058 (T), 740 (MC), 594 (Mf/DC), 201 (B) single cells) (PC = Plasma cell, MC = Mast cell, Mf/DC = Macrophage/Dendritic cell) **e** Flowcytometric antibody intensity (maximum signal) proportion plot and **f** the mRNA expression of canonical markers distribution of Ctrl, CeD and GFD cells from LP. **g** Histogram of the mean expression across the single cells with drop-out genes in pink. **h** Histogram of the noise/signal expression ratios across the genes. **i** Scatter plots depicting the breakdown of the single cells across the donors projected on the UMAP clusters (Fig. 1b). Source data for panels **a** and **b** are provided as a Source Data file.

Supplementary Fig. 2

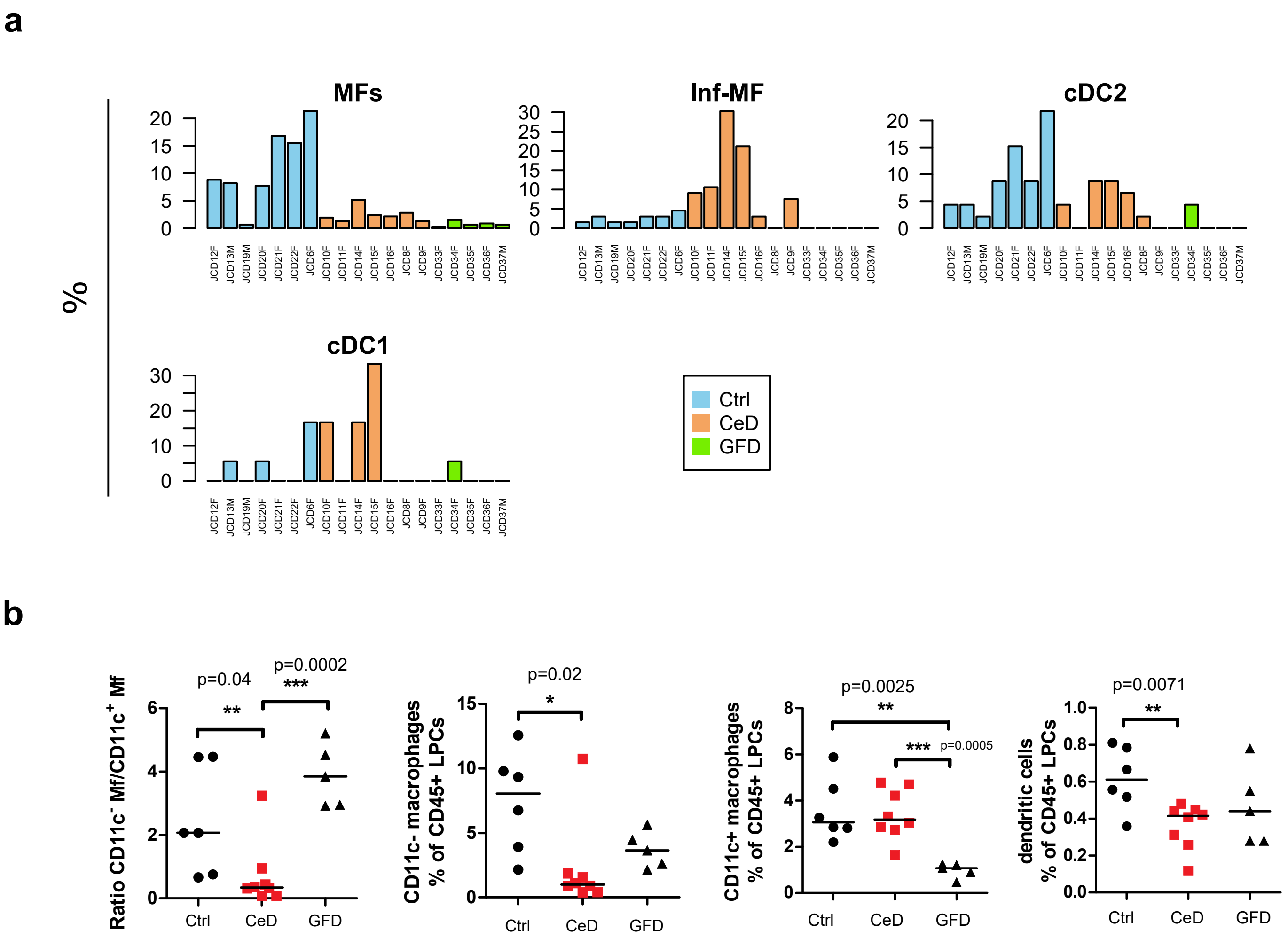

Supplementary Fig. 2 | Single-cell landscape of MF-DC cells in human small intestine in Ctrl, CeD and GFD samples.

a Bar plots showing the contribution of individual donors to each myeloid cluster (Fig. 2a) (MF = Macrophage, Inf-MF = Inflammatory Macrophage, cDC = classical Dendritic cell; Ctrl = control subjects, CeD = Celiac disease subjects, GFD = Celiac patients on gluten-free diet). **b** Scatter plots of the ratios of the CD11c^{+}/- Mfs and also DCs among the total CD45^{+} cells (n = 6 (Ctrl), 8 (CeD), 5 (GFD) donors) (two-tailed Student's t-test p values, * < 0.05, ** < 0.01, *** < 0.001); Source data are provided as a Source Data file.

Supplementary Fig. 3

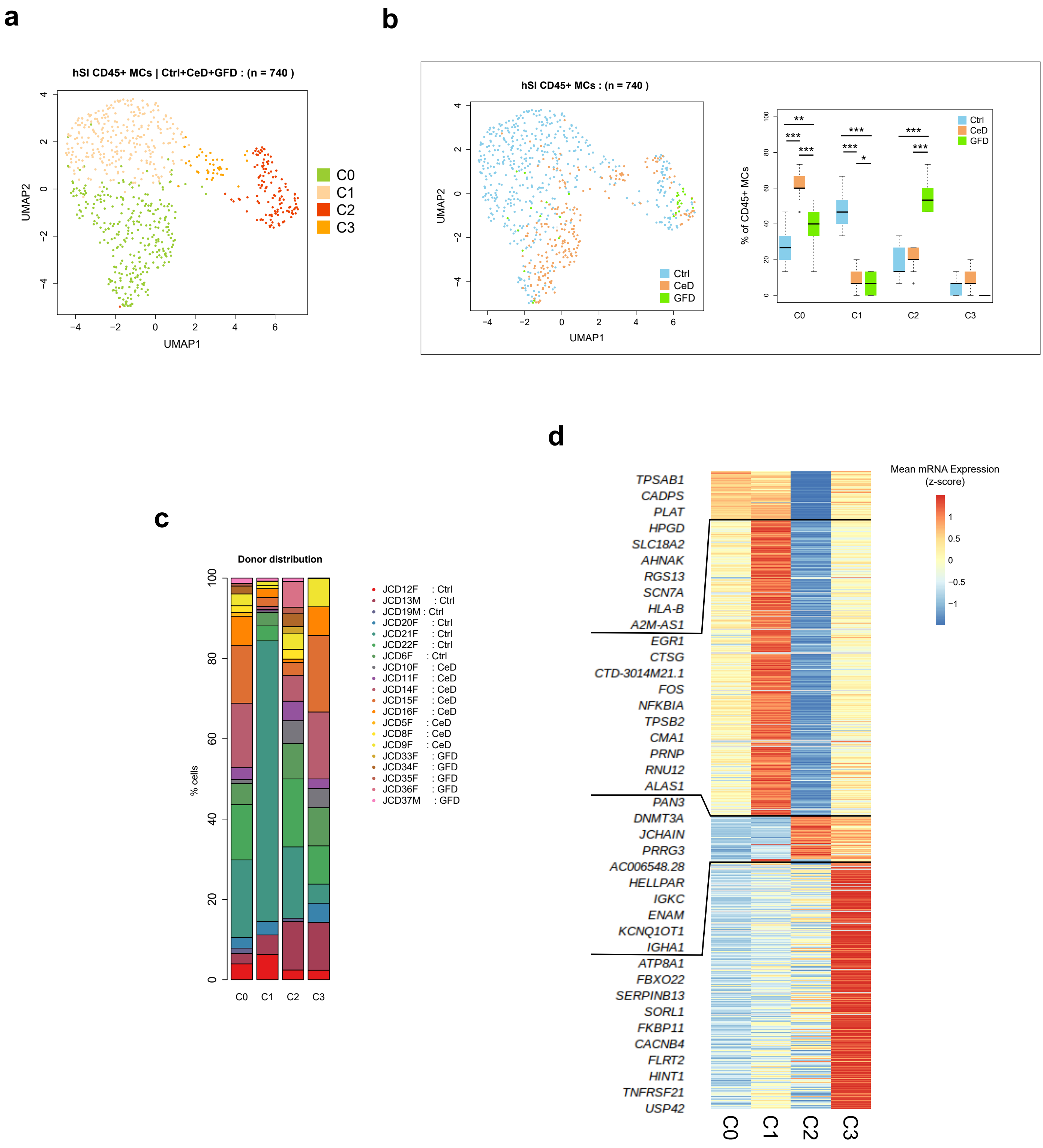

Supplementary Fig. 3 | Single-cell landscape of Mast cells in human small intestine in Ctrl, CeD and GFD samples.

a UMAP clustering plot of $CD45^+$ Mast cells (MCs) in Ctrl (control subjects), CeD (Celiac disease patients) and GFD (Celiac patients on gluten-free diet) samples. **b** Scatter plot (left panel) showing the projection of mast cells from Ctrl, CeD and GFD conditions on the UMAP clustering and boxplots (right panel) depicting the percentage of the cells among the total number of obtained $CD45^+$ mast cells across the UMAP clusters from Ctrl, CeD and GFD samples (center line, median; box limits, upper and lower quartiles; whiskers, minimum and maximum values; points, outliers, 1.5x interquartile range; two-sided t-test p-value $<=$ 0.05 (C0: Ctrl vs CeD = 1.89×10^{-7}, Ctrl vs GFD = 0.0088, CeD vs GFD = 5.71×10^{-6}; C1: Ctrl vs CeD = 5.02×10^{-6}, Ctrl vs GFD = 2.64×10^{-6}, CeD vs GFD = 0.0191; C2: Ctrl vs GFD = 1.00×10^{-8}, CeD vs GFD = 5.60×10^{-8}); n = 10 randomized cell subsampling). **c** Bar plots showing the contribution of each donor per each cluster of mast cells. **d** Heatmap plot presenting the mean expression of the top differentially expressed genes per each cluster of mast cells.

Supplementary Fig. 4

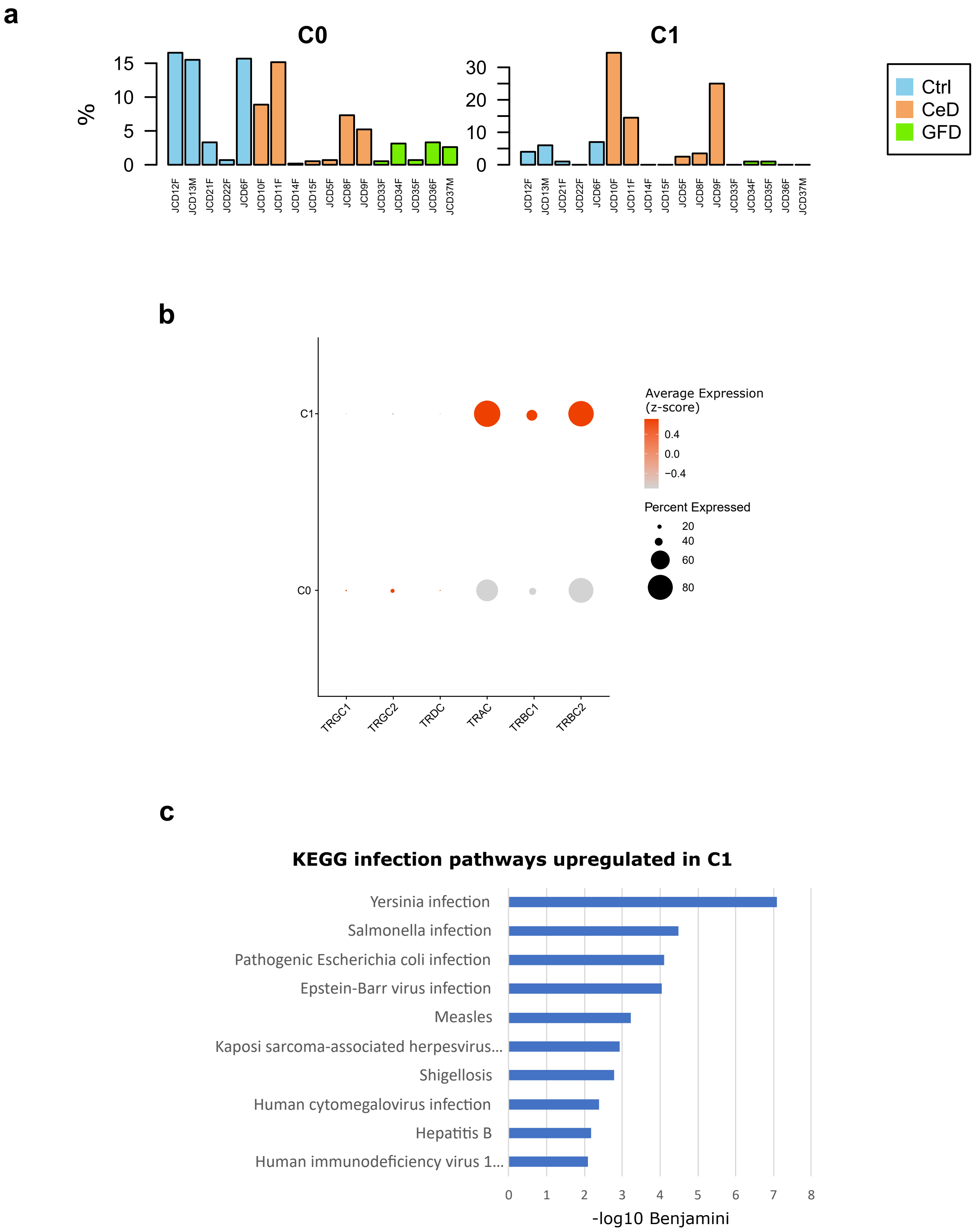

Supplementary Fig. 4 | Single-cell landscape of $CD4^+$ T cells in Lamina Propria of human small intestine in Ctrl, CeD and GFD samples.

a Bar plots of donor contribution per each $CD4^+$ T-cell cluster (Fig. 4a) (Ctrl = control subjects, CeD = Celiac disease subjects, GFD = Celiac patients on gluten-free diet). **b** Dot plot showing the mRNA expression of TCR constant region genes in UMAP clusters. **c** Bar plots showing the top infection-related KEGG pathways in CD4 C1 cells.

Supplementary Fig. 5

a

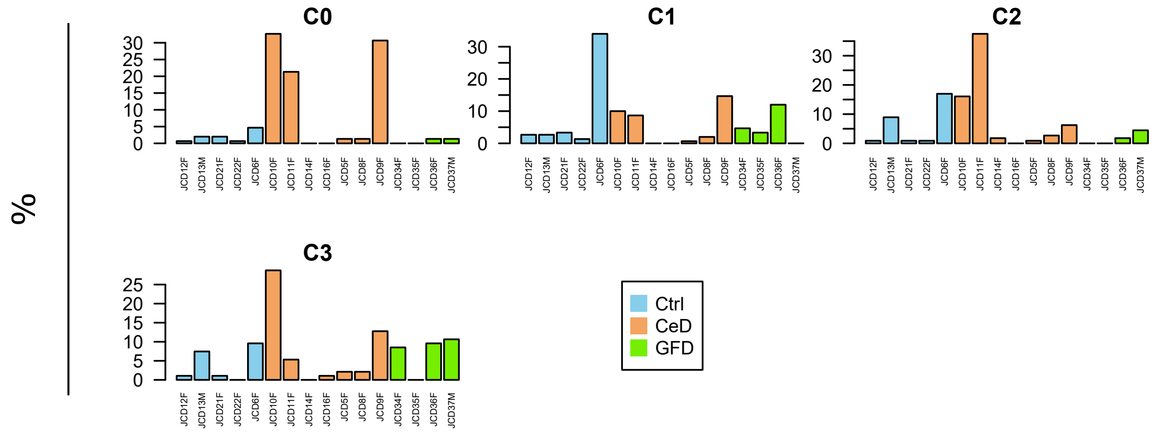

b

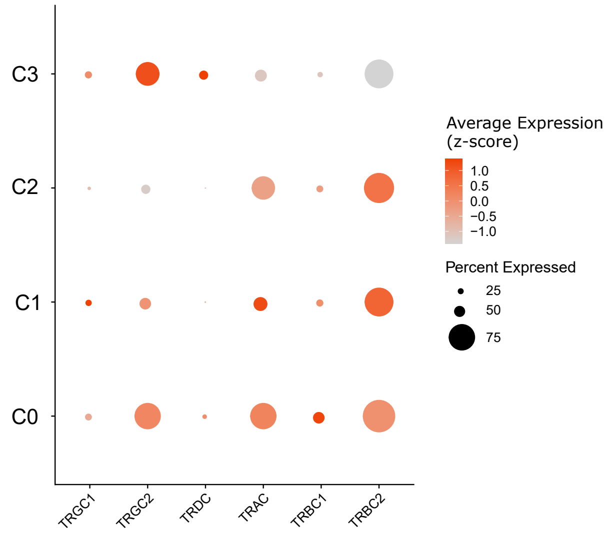

Supplementary Fig. 5 | Single-cell landscape of $CD8^+$ T cells in Lamina Propria of human small intestine in Ctrl, CeD and GFD samples.

a Bar plots showing the contribution of each donor cells per each cluster (Fig. 5a) (Ctrl = control subjects, CeD = Celiac disease subjects, GFD = Celiac patients on gluten-free diet).

b Dot plot depicting the mRNA expression of TCR genes in the $CD8^+$ T cells in the UMAP clusters.

Supplementary Fig. 6

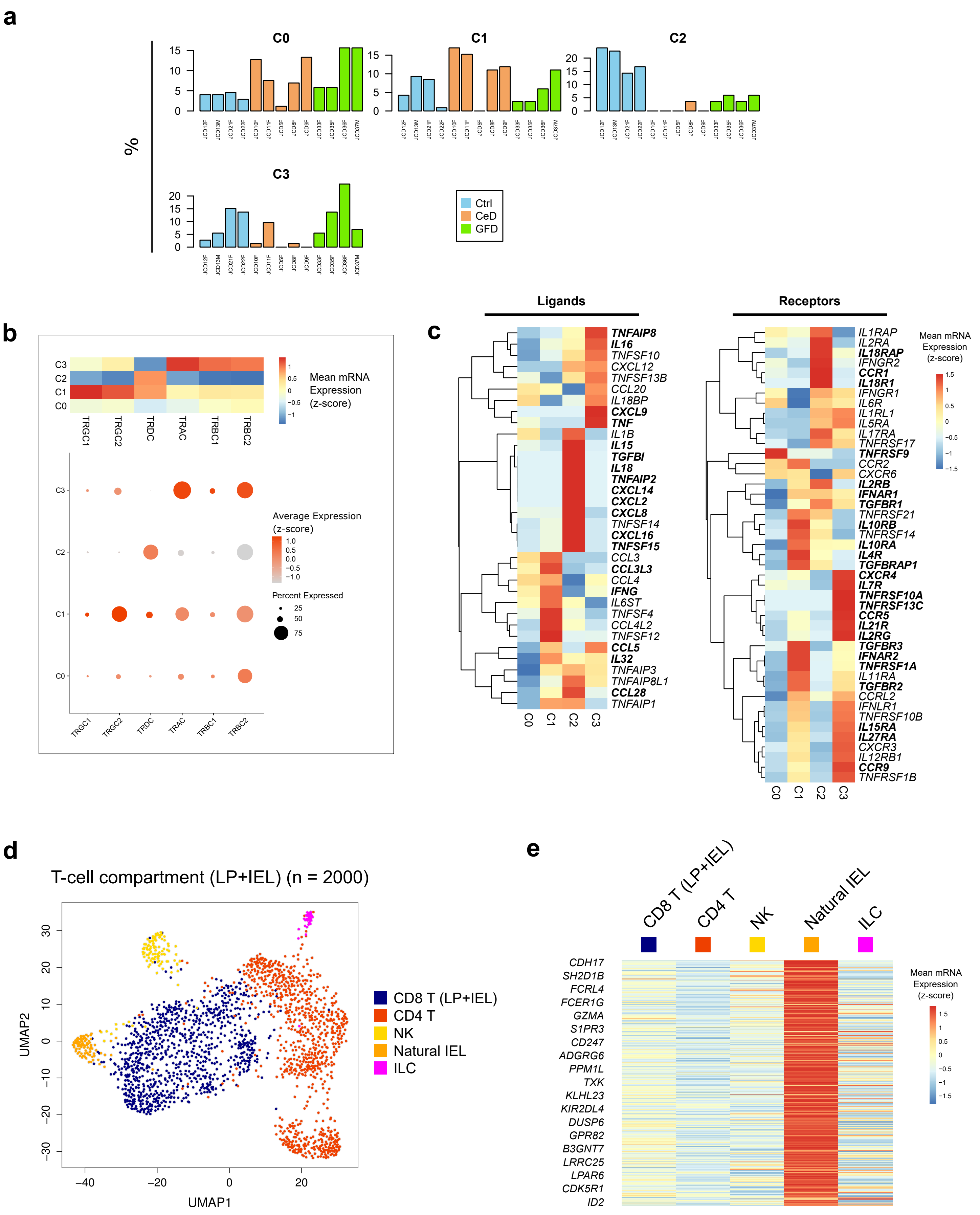

Supplementary Fig. 6 | Single-cell landscape of CD45$^+$ Intraepithelial lymphocytes of human small intestine in Ctrl, CeD and GFD samples.

a Bar plots showing the contribution of different donors to each Intraepithelial lymphocytes (IELs) cluster (Fig. 6a) (Ctrl = control subjects, CeD = Celiac disease subjects, GFD = Celiac patients on gluten-free diet). **b** Dot plot (bottom) depicting the mRNA expression of TCR genes in the IELs in the UMAP clusters with heatmap of the mean expression of the TCR genes across the clusters (top). **c** Heatmap plots of gene expression of cytokine ligands (left) and receptors (right) between the clusters of IELs (differentially expressed genes per each cluster are shown in bold). **d** UMAP plot showing the clustering of T-cell compartment cells from Lamina Propria (LP) in relation to natural IEL (NK = Natural Killer cells, ILC = Innate Lymphoid Cells). **e** Heatmap plot depicting the natural IEL specific gene signature (top genes) across all the IEL and LP T-compartment cells (empirical Bayes two-sided p-value $\leq$ 0.05).

Supplementary Fig. 7

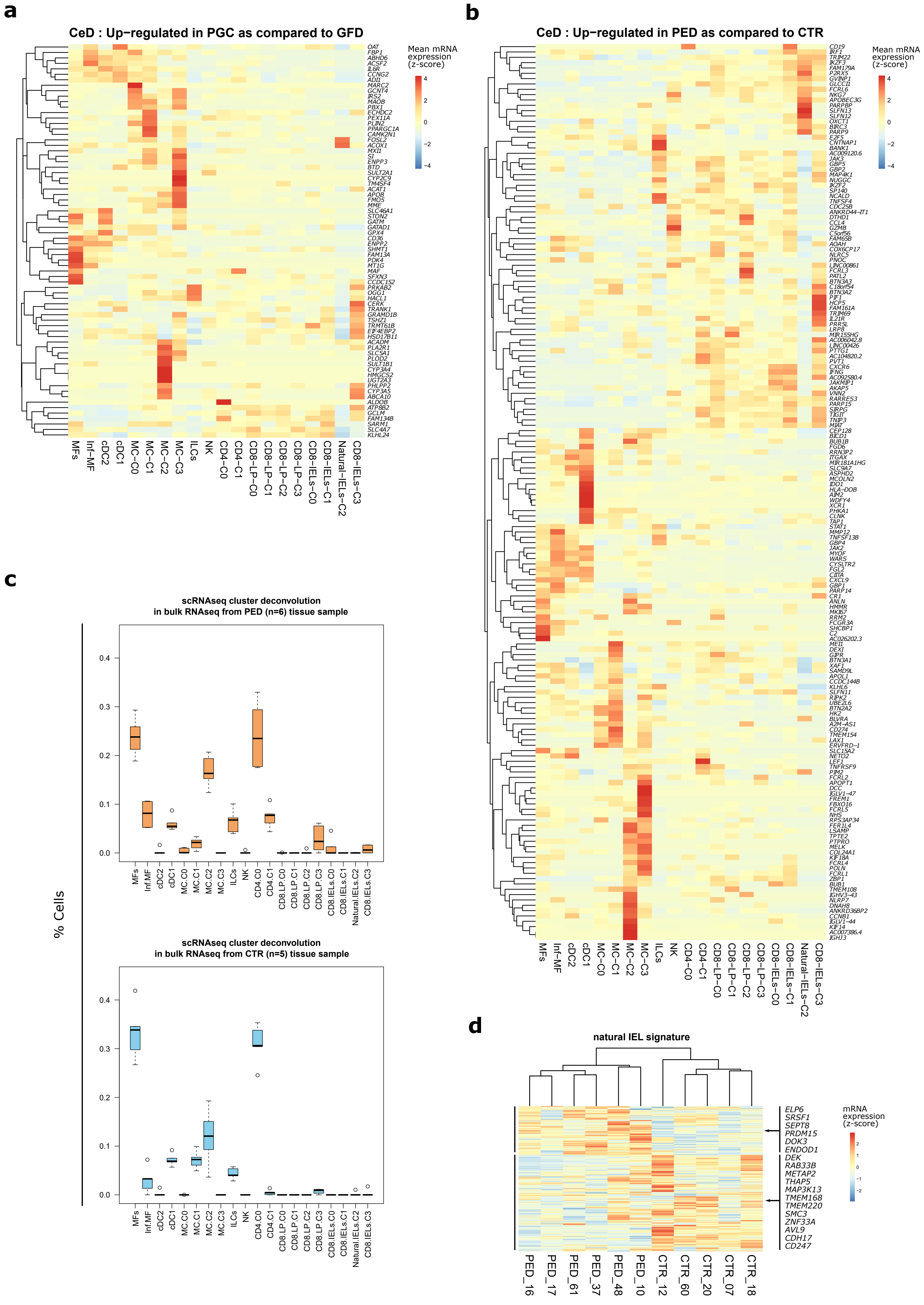

Supplementary Fig. 7 | Comparison analysis of the single-cell signatures of $CD45^+$ immune cells of human small intestine in Ctrl, CeD and GFD samples with bulk RNAseq data.

a Heatmap expression plot of the upregulated gene signature upon post-gluten challenge obtained from *Dotsenko* dataset across the single-cell clusters identified in CeD samples in our dataset (Ctrl = control subjects, PGC = Post gluten challenge subjects, GFD = Celiac patients on gluten-free diet). **b** Heatmap expression plot of the upregulated gene signature in Celiac (PED) as compared to control (CTR) obtained from *van der Graaf* dataset across the single-cell clusters identified in CeD (Celiac disease) subjects in our dataset. **c** Boxplots resulted from deconvolution analysis showing the proportion of the single-cell clusters in bulk RNAseq dataset (*van der Graaf*) (center line, median; box limits, upper and lower quartiles; whiskers, minimum and maximum values; points, outliers, 1.5x interquartile range; n = 5 CTR and 6 PED). **d** Heatmap expression plot of our identified natural IEL signature in bulk RNAseq dataset (*van der Graaf*).

Supplementary Fig. 8

a

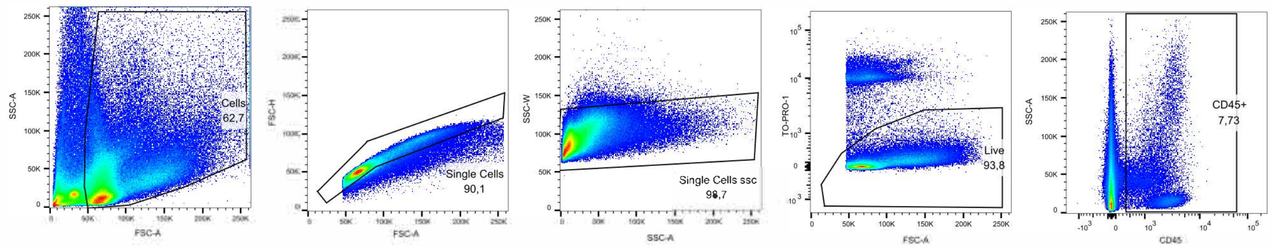

b

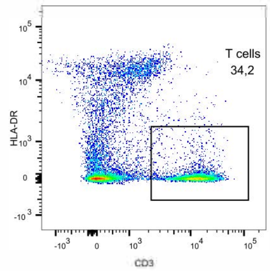

c

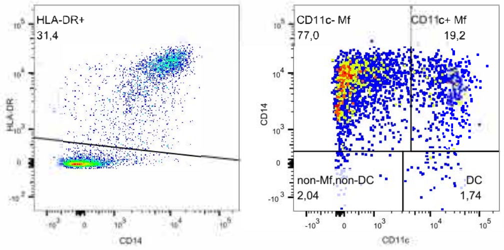

d

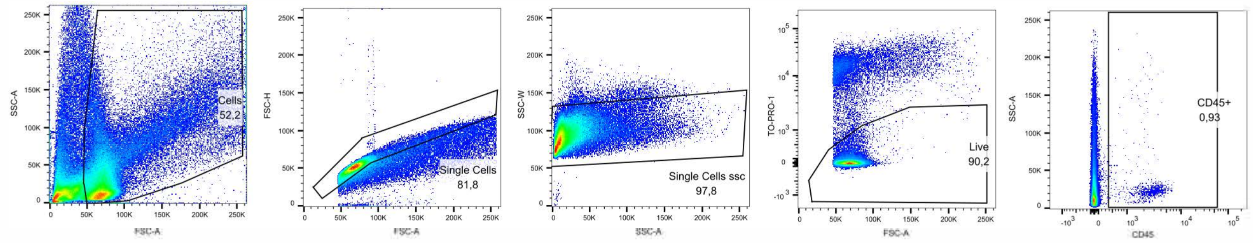

Supplementary Fig. 8 | Flowcytometry gating strategy of the immune cells in the human small intestine.

a Scatter plots depicting the gating strategy of the $CD45^+$ immune cell sorting from the Lamina Propria (LP) of the human small intestine (corresponding to Fig. 1a and Supplementary Fig. 1a). **b** Scatter plots showing the assignment of $CD3^+$ T (corresponding to Supplementary Fig. 1b) and **c** CD11c+/- macrophage (Mf) subsets and dendritic cells (DC) (corresponding to Supplementary Fig. 2b) from the LP of the human small intestine. **d** Scatter plots showing the gating strategy of $CD45^+$ intraepithelial lymphocytes (IELs) sorting from the human small intestine (corresponding to Fig. 6).

Supplementary Table 1: Metadata of the subjects recruited in this study

Sample	Age (yrs)	Sex	Status	Marsh score	IgA anti-tTG	Diagnosis
JCD5F	20-30	F	CeD	3B	> 120 U/ml	CeD
JCD6F	30-40	F	ctrl	-	1 U/ml	IBS
JCD8F	50-60	F	CeD	3A	39 U/ml	CeD
JCD9F	30-40	F	CeD	3C	> 120 U/ml	CeD
JCD10F	30-40	F	CeD	3B/3C	110 U/ml	CeD
JCD11F	70-80	F	CeD	3B	> 120 U/ml	CeD
JCD12F	40-50	F	ctrl	-	< 1 U/ml	Dyspepsia
JCD13M	30-40	M	ctrl	-	< 1 U/ml	Meteorism
JCD14F	10-20	F	CeD	3C	> 120 U/ml	CeD
JCD15F	20-30	F	CeD	3C	34 U/ml	CeD
JCD16F	20-30	F	CeD	3B	60 U/ml	CeD
JCD19M	40-50	M	Ctrl	-	n.a.	n.a.
JCD20F	40-50	F	ctrl	-	< 1 U/ml	Abdominal symptoms
JCD21F	30-40	F	ctrl	-	< 1 U/ml	IBS
JCD22F	10-20	F	ctrl	-	< 1 U/ml	IBS
JCD33F	30-40	F	GFD	0	n.a.	CeD
JCD34F	40-50	F	GFD	0	n.a.	CeD
JCD35F	30-40	F	GFD	0	n.a.	CeD
JCD36F	30-40	F	GFD	0	n.a.	CeD
JCD37M	40-50	M	GFD	0	n.a.	CeD

Supplementary Table 2 : Flow cytometry antibodies/reagents used in this study

**Antibody/Reagent**	**Clone**	**Vendor**	**Catalogue number**	**Lot number**	**Dilution**
FcR Blocking Reagent		Miltenyi Biotec	130-059-901	5181017651	1:10
CD45-APC-H7	2D1	BD Biosciences	560274	B265819	1:20
CD3-APC	OKT3	Biolegend	317318	B281030	1:20
CD19-BV421	HIB19	Biolegend	302234	B318042	1:20
HLA-DR-PerCP-Cy5.5	L243	Biolegend	307630	B293514	1:20
CD14-PE-Cy7	HCD14	Biolegend	325618	B262300	1:20
CD11c-PE	S-HCL-3	BD Biosciences	333149	8220676	1:20
EpCAM-FITC	Ber-EP4	Dako	F0860	41294336	1:10
CD27-BV605	O323	Biolegend	302830	B244220	1:20
CD103-BV605	Ber-ACT8	Biolegend	350218	B241238	1:20
To-Pro-1-iodide		ThermoFisher	T3602		10 μM
