# Supplementary material for: Single cell transcriptomic analysis of the immune cell compartment in the human small intestine and in Celiac disease
Source: Nat Commun. 2022 Aug 22;13:4920. doi: 10.1038/s41467-022-32691-5 (PMC9395525; doi:10.1038/s41467-022-32691-5)
Supplement: Supplementary file 3 — Description of Additional Supplementary Files [file 41467_2022_32691_MOESM3_ESM.pdf]

Description of Additional Supplementary Files

Supplementary Data 1: Tables of differentially expressed genes between the cell clusters and their related gene ontology analysis tables related to each figure.
