# Supplementary material for: Single cell transcriptomic analysis of the immune cell compartment in the human small intestine and in Celiac disease
Source: Nat Commun. 2022 Aug 22;13:4920. doi: 10.1038/s41467-022-32691-5 (PMC9395525; doi:10.1038/s41467-022-32691-5)
Supplement: Supplementary file 5 — Reporting Summary [file 41467_2022_32691_MOESM5_ESM.pdf]

nature portfolio

Corresponding author(s): Hendrik G. Stunnenberg

Last updated by author(s): Apr 28, 2022

Reporting Summary

Nature Portfolio wishes to improve the reproducibility of the work that we publish. This form provides structure for consistency and transparency in reporting. For further information on Nature Portfolio policies, see our Editorial Policies and the Editorial Policy Checklist.

Statistics

For all statistical analyses, confirm that the following items are present in the figure legend, table legend, main text, or Methods section.

n/a	Confirmed	
☐	☒	The exact sample size (n) for each experimental group/condition, given as a discrete number and unit of measurement
☐	☒	A statement on whether measurements were taken from distinct samples or whether the same sample was measured repeatedly
☐	☒	The statistical test(s) used AND whether they are one- or two-sided<br>*Only common tests should be described solely by name; describe more complex techniques in the Methods section.*
☐	☒	A description of all covariates tested
☒	☐	A description of any assumptions or corrections, such as tests of normality and adjustment for multiple comparisons
☐	☒	A full description of the statistical parameters including central tendency (e.g. means) or other basic estimates (e.g. regression coefficient) AND variation (e.g. standard deviation) or associated estimates of uncertainty (e.g. confidence intervals)
☐	☒	For null hypothesis testing, the test statistic (e.g. F, t, r) with confidence intervals, effect sizes, degrees of freedom and P value noted<br>*Give P values as exact values whenever suitable.*
☒	☐	For Bayesian analysis, information on the choice of priors and Markov chain Monte Carlo settings
☒	☐	For hierarchical and complex designs, identification of the appropriate level for tests and full reporting of outcomes
☒	☐	Estimates of effect sizes (e.g. Cohen's d, Pearson's r), indicating how they were calculated

Our web collection on statistics for biologists contains articles on many of the points above.

Software and code

Policy information about availability of computer code

Data collection	For cell sorting, FACS Diva software version 8 (BD Biosciences) was used.
Data analysis	Flowcytometry data analysis was performed using FlowJo software v 10.6 and Prism 6.0 (GraphPad Software).<br>The raw FASTQ files were used as input for the CEL-Seq2 pipeline (Hashimshony, T. et al. 2016) however, incorporating STAR aligner (v 2.5.2b) (https://github.com/alexdobin/STAR) to map the sequenced reads against the human genome HG38 using the default parameters.<br>The demultiplexed, uniquely mapped reads per each single cell were counted against the reference genome and PCR deduplicated using HTSeq-count (v 0.7.2) part of the CEL-Seq2 pipeline and the table of read counts for all single cells was built using R environment v3.4.<br>The dataset was normalized using size-factor calculations within SCRAN package (v 1.4.5) incorporating the ERCC spike-ins.<br>We used Seurat v.3 pipeline (Butler et al. 2018) to identify the possible clusters of cells and the main cell populations.<br>For gene ontology analysis we used ClusterProfiler (v 4) pipeline with default parameters and for heatmap visualisation we used pheatmap package (v 1.0.12) within R environment.<br>Bayesian statistics was performed using Limma package (Ritchie ME et al. 2015) from Bioconductor v3.6 with the default parameters.<br>For deconvolution analysis we used SCDC pipeline (v 0.0.0.9000) with default parameters.

For manuscripts utilizing custom algorithms or software that are central to the research but not yet described in published literature, software must be made available to editors and reviewers. We strongly encourage code deposition in a community repository (e.g. GitHub). See the Nature Portfolio guidelines for submitting code & software for further information.

Data

Policy information about availability of data

All manuscripts must include a data availability statement. This statement should provide the following information, where applicable:

- Accession codes, unique identifiers, or web links for publicly available datasets
- A description of any restrictions on data availability
- For clinical datasets or third party data, please ensure that the statement adheres to our policy

The sequencing data generated in this study has been deposited at the European Genome-phenome Archive (EGA), which is hosted by the EBI and the CRG, under accession number EGAS00001003751 [https://ega-archive.org/studies/EGAS00001003751]. The Source Data file containing the raw data is provided as a supplementary file.

Field-specific reporting

Please select the one below that is the best fit for your research. If you are not sure, read the appropriate sections before making your selection.

- [x] Life sciences
- [ ] Behavioural & social sciences
- [ ] Ecological, evolutionary & environmental sciences

For a reference copy of the document with all sections, see nature.com/documents/nr-reporting-summary-flat.pdf

Life sciences study design

All studies must disclose on these points even when the disclosure is negative.

Sample size	No sample size calculation was performed. In this study, we aimed to collect as many tissue samples as possible from patients undergoing evaluation for CeD during the project period. 8 (all females, median age 29.7) patients with confirmed untreated CeD (Marsh score 3A-3C) and 7 (6 females, median age 35.8) confirmed non-CeD controls and 5 patients (4 female, median age 35) on gluten-free diet (Marsh score 0) have been enrolled in the study.
Data exclusions	To remove the low-quality cells from the dataset, we applied several criteria. Cells with MAD value of ERCC, mitochondrial counts and library size greater than 3 were removed using SCRAN package (v 1.4.5). Next, cells with total detected genes less than 300 and more than 4000 were assigned as outliers.<br>We also removed the drop-out genes from the dataset; for this we removed the low-abundance genes with either zero expressions for all the cells or the genes with mean expression value below the threshold on the distribution model or the genes with high background noise to signal ratio.
Replication	For celiac samples, we isolated the target cells from 8 independent patients; for controls we isolated the target cells from 7 independent individuals and for GFD samples we isolated the target cells from 5 independent patients. The cell annotation and cluster analysis confirmed that, beside subtle donor-to-donor variations within the identified clusters of cells, the identified cell populations and their related gene signatures from different donors were reproducible between the donors/conditions.
Randomization	Randomization is not relevant to this study, as subjects were allocated to experimental groups based on clinical workup. Diagnosis of CeD followed standard procedure (Ludvigsson et al., 2014). patients with confirmed untreated CeD (Marsh score 3A-3C) and confirmed non-CeD controls and patients on gluten-free diet for at least one year (Marsh score 0) have been enrolled in the study.
Blinding	The sample collection was performed without group allocation blinding and the investigators were aware of each collected sample/condition during subject recruitment, tissue processing and cell isolation. However, the collected samples were given generic codes for RNA sequencing experiments and the investigator was blinded to the sample's condition during the library preparation and sequencing.

Reporting for specific materials, systems and methods

We require information from authors about some types of materials, experimental systems and methods used in many studies. Here, indicate whether each material, system or method listed is relevant to your study. If you are not sure if a list item applies to your research, read the appropriate section before selecting a response.

Materials & experimental systems

n/a	Involved in the study
[ ]	[x] Antibodies
[x]	[ ] Eukaryotic cell lines
[x]	[ ] Palaeontology and archaeology
[x]	[ ] Animals and other organisms
[ ]	[x] Human research participants
[x]	[ ] Clinical data
[x]	[ ] Dual use research of concern

Methods

n/a	Involved in the study
[x]	[ ] ChIP-seq
[ ]	[x] Flow cytometry
[x]	[ ] MRI-based neuroimaging

Antibodies

Antibodies used

Antibody/Reagent;Clone;Vendor;Catalogue number;Lot number;Dilution
FcR Blocking Reagent;;Miltenyi Biotec;130-059-901;5181017651;1:10
CD45-APC-H7 ;2D1;BD Biosciences;560274;B265819;1:20
CD3-APC;OKT3;Biolegend;317318;B281030;1:20
CD19-BV421;HIB19;Biolegend;302234;B318042;1:20
HLA-DR-PerCP-Cy5.5;L243;Biolegend;307630;B293514;1:20
CD14-PE-Cy7 ;HCD14;Biolegend;325618;B262300;1:20
CD11c-PE ;S-HCL-3;BD Biosciences;333149;8220676;1:20
EpCAM-FITC;Ber-EP4;Dako;F0860;41294336;1:10
CD27-BV605;O323;Biolegend;302830;B244220;1:20
CD103-BV605 ;Ber-ACT8;Biolegend;350218;B241238;1:20
To-Pro-1-iodide;;ThermoFisher;T3602;;10 mM

Validation

The commercial antibodies were validated by the manufacturer. Biolegend: In an on-going effort to increase the confident use of our antibodies across all assay platforms, BioLegend has been undertaking a program of extensive antibody validation of existing clones using chromogenic methods like DAB amplification or detection with fluorophores commonly used.
BD Bioscience: The specificity is confirmed using multiple applications including a combination of flow cytometry, immunofluorescence or western blot and the antibodies are titrated on the relevant positive or negative cells and the pre-titrated test size at an optimal concentration, commercially available tubes are provided for researchers.

Human research participants

Policy information about studies involving human research participants

Population characteristics

8 (all females, median age 29.7) patients with confirmed untreated CeD (Marsh score 3A-3C) and 7 (6 females, median age 35.8) confirmed non-CeD controls and 5 patients (4 female, median age 35) on gluten-free diet (Marsh score 0) were enrolled

Recruitment

Duodenal biopsies were obtained during routine endoscopy at Akershus University Hospital and Oslo University Hospital from patients referred due to suspicion of CeD. We did not observe any potential self-selection bias or other biases in the recruitment process.

Ethics oversight

The study was approved by the Norwegian Regional Committee for Medical Research ethics (REK 20521/6544)

Note that full information on the approval of the study protocol must also be provided in the manuscript.

Flow Cytometry

Plots

Confirm that:

- [x] The axis labels state the marker and fluorochrome used (e.g. CD4-FITC).
- [x] The axis scales are clearly visible. Include numbers along axes only for bottom left plot of group (a 'group' is an analysis of identical markers).
- [x] All plots are contour plots with outliers or pseudocolor plots.
- [x] A numerical value for number of cells or percentage (with statistics) is provided.

Methodology

Sample preparation

To separate the epithelium and IELs, biopsies were shaken twice in 6.5 ml of PBS with 2mM EDTA (Sigma-Aldrich), 1% FCS (Sigma-Aldrich) and 1µM flavopirydol (Sigma-Aldrich) for 10 min at 37°C. Supernatants containing the epithelial fractions were combined, washed, passed through 100 µm cell strainers (Miltenyi Biotec), washed again and kept on ice until staining. Epithelium-free mucosa was minced and incubated with stirring in 2.5 ml of RPMI1640 (Lonza) containing 10% FCS, 1% Pen/

	Strep (Lonza), 1µM flavopiridol77, 0.25 mg/ml Liberase TL (Roche) and 20 U/ml DNase I (Sigma) for 40 min at 37°C. Halfway through the incubation the samples were triturated using 1 ml pipette to facilitate complete digestion. Digested cell suspension was passed through 100 µm cell strainer and washed twice.
Instrument	FACS Aria IIu or FACS Aria III
Software	FACS Diva software version 8 (BD Biosciences). FlowJo software version 10.6
Cell population abundance	Around 1000 single cells per donor were sorted in 384-well plates after gating strategy.
Gating strategy	Doublets were identified and excluded based on scatter parameters in forward scatter area versus height (FSC-A/FSC-H) and side scatter area versus width (SSC-A/SSC-W) plots. Cells of interest (single live CD45+, or single live CD45+(CD27-)CD3-CD19- to enrich for myeloid cells) were index sorted (48 epithelial cells per patient and the rest LP cells) into Bio-Rad Hard Shell 384 well microplates (Bio-Rad)

☒ Tick this box to confirm that a figure exemplifying the gating strategy is provided in the Supplementary Information.
